# Two Types of Management for the Noninvasive Treatment of Pectus Excavatum in Neonatal Puppies—Case Reports

**DOI:** 10.3390/ani13050906

**Published:** 2023-03-02

**Authors:** Keylla Helena Nobre Pacífico Pereira, Kárita da Mata Fuchs, Lara Ataídes Arantes Terçariol, Renata Cesar Silva, Gabriel de Azevedo Camargo, Júlia Cosenza Mendonça, Netelin Tainara Paulino, Marcelo Alejandro Zone, Eunice Oba, Maria Lucia Gomes Lourenço

**Affiliations:** 1 Veterinary Neonatology Research Group, Department of Veterinary Clinic, School of Veterinary Medicine and Animal Science, São Paulo State University (UNESP), Botucatu 18618-681, Brazil; 2 Self-Employed Veterinarian, Rio Verde 75907-453, Brazil; 3 Self-Employed Veterinarian, Bauru 17022-152, Brazil; 4 Self-Employed Veterinarian, 43882 Barcelona, Spain; 5 Department of Veterinary Surgery and Animal Reproduction, School of Veterinary Medicine and Animal Science, São Paulo State University (UNESP), Botucatu 18618-681, Brazil

**Keywords:** congenital malformation, dyspnea, splint, bandage, thorax, dog, brachycephalic breeds

## Abstract

**Simple Summary:**

Congenital malformations in newborn dogs are still largely poorly understood and are inadequately treated, which leads to high mortality rates in these patients. This report describes the clinical signs, diagnosis and two types of management for the treatment of pectus excavatum in puppies, a malformation of the chest wall that can lead to progressive cardiorespiratory changes and progression to death. This case study assists veterinarians in the clinical approach of pectus excavatum to perform adequate medical conduct and ensure greater neonatal survival.

**Abstract:**

Pectus excavatum is a deformity of the thorax characterized by ventrodorsal narrowing of the sternum bone and costal cartilages, which can lead to compression and cardiopulmonary alterations in dogs, presenting a high prevalence in brachycephalic breeds. The aim of this report was to describe two types of management for the noninvasive treatment of pectus excavatum in newborn puppies of the breeds French Bulldog and American Bully. The puppies presented dyspnea, cyanosis and substernal retraction during inspiration. The diagnosis was performed by physical examination and confirmed by chest X-ray. Two types of splints were performed (a circular splint with plastic pipe and a paper box splint on the chest), aiming at thoracic lateral compression and frontal chest remodeling. The management was effective for the conservative treatment of mild-grade pectus excavatum, resulting in the repositioning of the thorax and improvement of the respiratory pattern.

## 1. Introduction

Pectus excavatum (excavated chest) is a congenital malformation of the development of the ventral chest wall, also known as the ‘funnel chest’ [1,2,3]. It is characterized by abnormal growth of the sternum and costal cartilages, where there is a deviation and ventrodorsal narrowing of the thorax, which presents with depression [1,3,4,5].

This deformity is described in humans, dogs, cats, lambs, calves, rabbits, and other species [1,6,7,8,9]. In canine species, the incidence of pectus excavatum is 0.33% [10], and pectus excavatum has a high prevalence in brachycephalic breeds, such as French Bulldog, English Bulldog, Pekingese, Pug, Maltese, Shih-Tzu and American Bully, associated with the probable genetic component [3,4]. In these breeds, pectus excavatum can be frequently diagnosed with other deformities, such as swimmer dog syndrome, and respiratory tract defects, such as nasal stenosis, tracheal hypoplasia, and soft palate hyperplasia (brachycephalic airway syndrome) [3,4,11,12,13].

Its etiology is not well defined, and it is believed to be a genetic predisposition with a hereditary component [6,14,15]. Some studies suggest shortening of the diaphragmatic tendon, congenital deficiency of the cranial muscles of the diaphragm and osteogenesis/abnormal chondrogenesis. However, the phenotype and a variety of conditions can also be associated, such as abnormalities in intrauterine pressure and a negative increase in intrathoracic pressure secondary to alterations in the upper airway (for example, airway obstruction from soft palate hyperplasia) [4,15,16].

The clinical manifestations of pectus excavatum are variable and are associated with pulmonary alterations secondary to reduced thoracic space or pulmonary atelectasis by cardiac compression (cardiac compression puts pressure on the lung, causing the pulmonary tissue to collapse with loss of volume). In some cases, they may present with recurrent pneumonia, causing chronic respiratory disease [6,15]. Changes in the cardiovascular system can be observed with deviation and compression of the heart, systolic or diastolic dysfunction, or related to other congenital cardiac alterations [3,4,15,17]. The main clinical signs observed are dyspnea, tachypnea, hyperpnea, pallor or the cyanosis of mucous membranes, intolerance to manipulation or exercise, apathy, lack of appetite, vomiting or regurgitation, cough, heart murmur and arrhythmias [9,12,15,17]; however, some animals may be asymptomatic. Depending on the severity of cardiorespiratory signs, this condition can lead to high mortality in affected dogs [12,18].

The diagnosis of pectus excavatum is made by physical examination, visualization and palpation of the depression formed in the sternum, which is confirmed by complementary exams, such as chest X-ray, computed tomography or magnetic resonance imaging.

**Figure 1:**
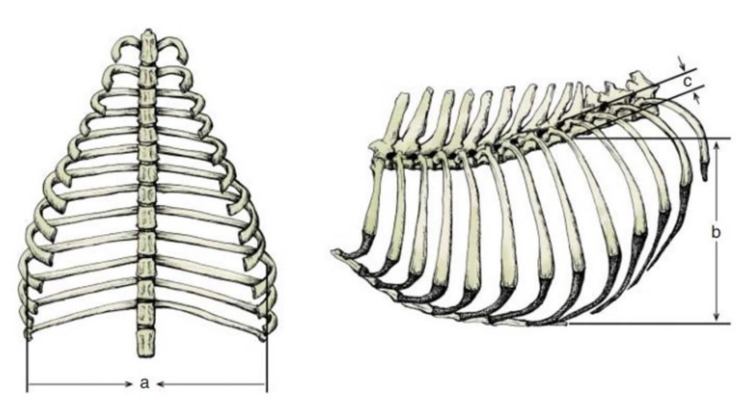
Frontosagittal and vertebral thoracic indices. The frontosagittal index is the ratio between the width of the thorax at the height of the tenth thoracic vertebra (**a**) and the distance between the center of the ventral surface of the tenth thoracic vertebra and the closest point to the sternum (**b**). The vertebral index is the ratio between the distance from the center of the dorsal surface of the selected vertebra to the nearest point of the sternum (**b**) and the dorsoventral diameter of the center of the same vertebra (**c**) (Adamovich-Rippie figure; Culp, 2016 [16]).

**Figure 2:**
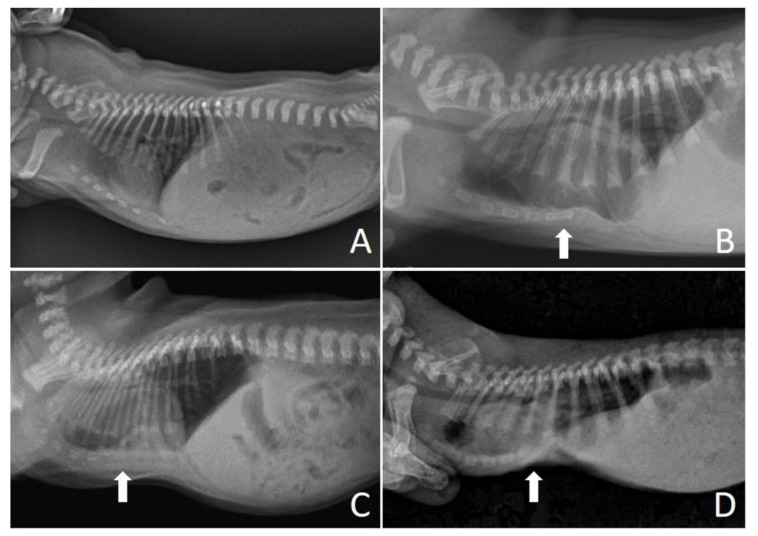
Chest X-rays of neonatal puppies of brachycephalic breeds. (**A**) Chest without alterations, indices—frontosagittal: 1.14 cm; vertebral: 13.4 cm. (**B**) Pectus excavatum of mild degree (arrow), indices—frontosagittal: 2 cm; vertebral: 11.25 cm. (**C**) Pectus excavatum of moderate degree (arrow), indices—frontosagittal: 3 cm; vertebral: 6 cm. (**D**) Severe pectus excavatum (arrow), indices—frontosagittal: 3.14 cm; vertebral: 5.8 cm.

The evaluation of pectus excavatum can be performed by measuring the frontosagittal and vertebral indices (Figure 1) on chest X-rays [15,16,19]. Table 1 shows the normal thoracic indices in dogs of nonbrachycephalic breeds, brachycephalic breeds and cats. Based on these indices, pectus excavatum can be classified as mild, moderate or severe [15,16,19] (Table 2). Figure 2 shows radiographs of neonatal puppies of brachycephalic breeds with normal thoraxes and with different degrees of pectus excavatum.

Initial treatment consists of clinical management of respiratory distress and therapy for respiratory infections. Treatment of pectus excavatum can be conservative or surgical, taking into account the age of the animal and the severity of the deformity. The change to conservative treatment can be performed for mild to moderate cases, and the use of a splint to compress the thorax has been described as an option for newborn puppies [1,2], and surgery is required for cases that do not respond to the splint, severe cases, or older animals [15,20]. In newborn pups, noninvasive treatment may be the first choice because constant bone growth allows the thorax to be reshaped quickly and effectively with corrective compression splints and bandages.

The aim of this report was to describe the clinical evolution of two cases of pectus excavatum in newborn puppies with two types of conservative treatment.

## 2. Case Reports

### 2.1. Case 1—Splint and Circular Bandage on Chest

A nine-day-old French Bulldog female from a kennel was treated at a veterinary clinic in Rio Verde, Brazil, with a 24 h history of respiratory distress. It was described by the tutor that from the beginning of dyspnea, the neonate presented with apathy, and he was reluctant to move, constantly vocalized and was in an orthopneic position. The newborn was fed exclusively breast milk and was kept with the other puppies on a mattress with blankets.

Regarding maternal information, the bitch was primiparous, at 18 months of age, fed a super-premium commercial diet, vaccinated, dewormed, with no history of diseases during the gestational phase, and supplemented with folic acid (0.1 mg/kg, once a day, for 30 days after insemination). No drugs were administered during the gestational phase. The delivery was eutocic, with the birth of seven puppies. No alterations were observed in the other neonates.

The kennel had a history of diagnosis of pectus excavatum in another litter of a bitch related to this mother. The kennel had 11 dogs. Two sister bitches (acquired for breeding with no history) that mated with different males had litters with pups showing pectus excavatum. In total, two litters and five puppies (one puppy from one litter and four from another) were diagnosed with this deformity. From this other litter, all four pups were also treated using the pipe splint; three survived, and one died.

On physical examination, the patient presented with marked dyspnea, substernal retraction during inspiration (Video S1), hyperpnea, cyanosis, pulmonary crackling, heart rate (HR) 196 bpm, respiratory rate (RF) 72 mpm, body temperature 36.3 °C, blood glucose 132 mg/dL, weight 356 g, absent suction and rooting reflexes, normohydrate, and no changes in cardiac auscultation. Thoracic deformity was observed on physical examination of the chest, with depression in the caudal region of the sternum bone and narrowing ventrodorsal direction.

In the outpatient treatment, oxygen therapy per mask with 40% oxygen was performed for two hours, and the bronchodilator aminofilin (24 mg/mL) was administered sublingually at a volume of 0.2 mL/100 g of weight.

After stabilization of the patient, complementary tests were performed: hemogram and chest X-ray. Moreover, 0.5 mL of blood from the jugular vein was collected for the realization of the hemogram, which demonstrated leukocytosis (26,000 leukocytes/μL) with left deviation associated with neutrophilia and slight absolute lymphocytosis.

**Figure 3:**
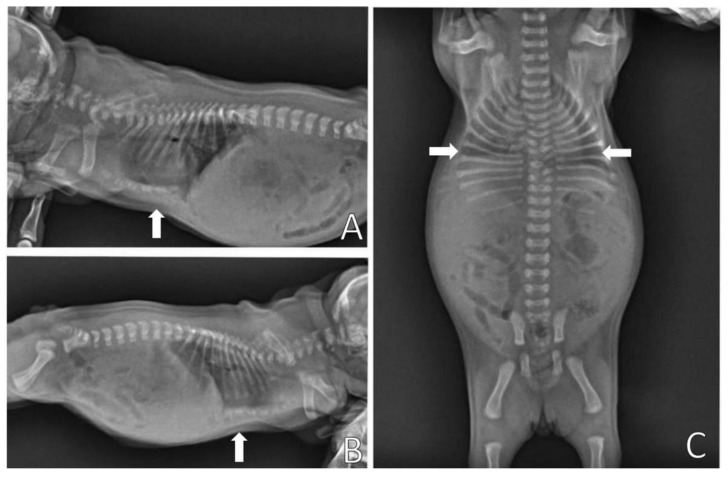
Chest X-ray. (**A**) Right side position and (**B**) left side position, demonstrating ventrodorsal deviation of the sternum bone (arrows); (**C**) Ventrodorsal position demonstrating narrowing of the thorax (arrows), pneumonia and left cardiac deviation.

On the chest X-ray (Figure 3), it was possible to observe the ventrodorsal deviation of the sternum in the medial and caudal region of the ventral thorax, diagnosing pectus excavatum. The frontosaginal and vertebral index corresponded to 1.8 cm (≤2.0 cm) and 10.2 cm (>9 cm), respectively, classifying the pectus as mild grade. In addition, cardiopulmonary alterations were found: pneumonia with alveolar pulmonary pattern, pulmonary hyperinflation, rounded cardiac silhouette (globose aspect) and cardiac deviation to the left.

**Figure 4:**
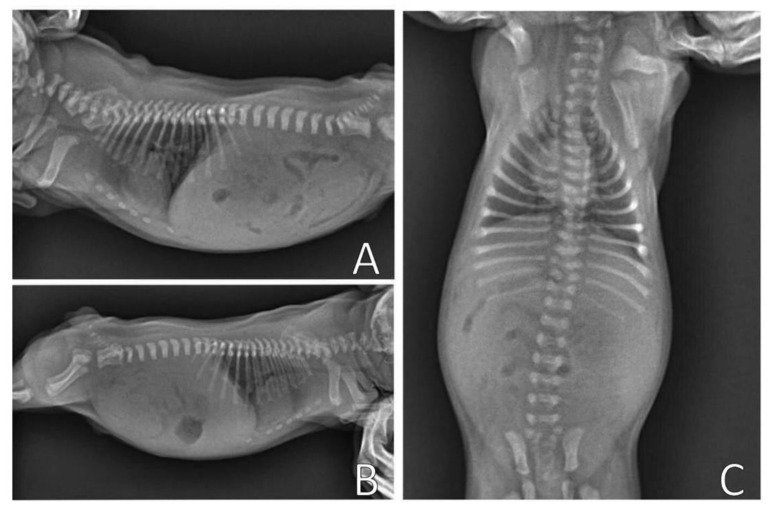
Normal thoracic radiographic examination. (**A**) Right side position; (**B**) Left side position; (**C**) Ventrodorsal position.

Figure 4 shows the chest X-ray of a neonate of the same litter, without thoracic alterations, to compare chest structures and aid in diagnosis.

Clinical treatment was initiated with antibiotic therapy (ceftriaxone 50 mg/kg subcutaneously every 12 h for five days); inhalation with a bronchodilator (aminophylline 24 mg/mL, in the volume of 0.2 mL/100 g of weight, every 12 h, for three days); N-acetylcistein 3 mg/kg orally every 8 h for five days; anti-inflammatory (fluticasona aerosol propionate 250 mcg, an intranasal spray every 8 h for five days), and feeding with commercial breast milk substitute (Support Milk Dog^®^) by orogastric tube (using a urethral probe number 04), while the neonate presented absent sucking reflex, at a volume of 3 mL/100 g of weight and temperature of 37 °C, every three hours.

**Figure 5:**
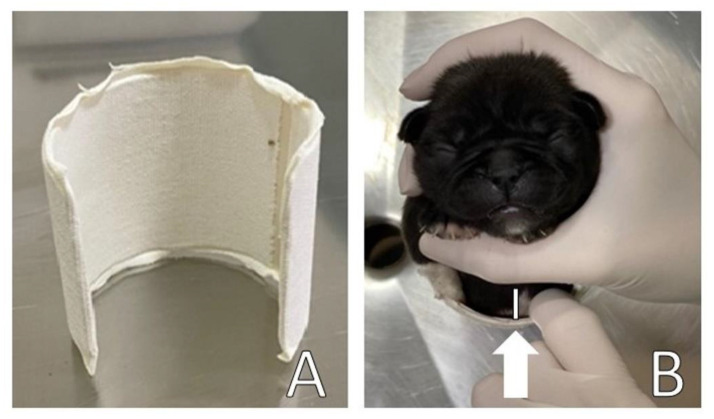
(**A**) PVC pipe used for making the splint; (**B**) Ventral space (2 cm) necessary for remodeling the thorax (arrow) and use of cotton to upholstery the sides of the splint.

A circular splint was made for the thorax (Figure 5A) in the form of a ‘C’ using a polyvinyl chloride (PVC) pipe [2] to perform slight lateral compression of the thorax for the remodeling of the rib cage and sternum bone. The width and length of the PVC corresponded to the width and length of the patient’s thorax. A ventral space (approximately 2 cm) was maintained (Figure 5B) to avoid ventral compression of the thorax on surfaces, allowing space for remodeling the thorax/sternum in the frontal direction.

**Figure 6:**
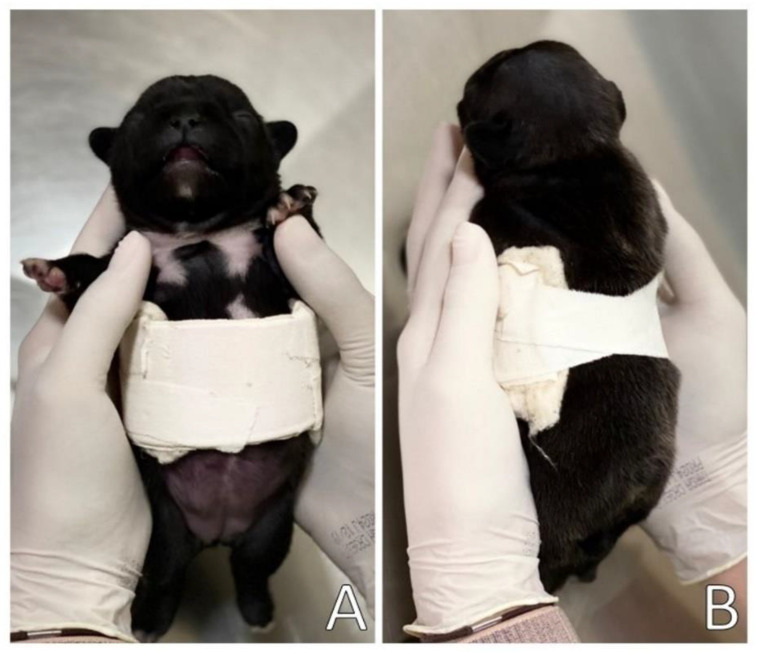
(**A**) Ventral view of the splint in the patient; (**B**) Dorsal view of the splint in the patient.

The splint was upholstered with cotton wool and medical tape on the sides to support and slightly compress the sides of the chest, as well as to avoid traumatic injuries to the patient, promoting comfort in a certain way. The splint was placed on the newborn’s chest, with the open part of the PVC on the patient’s back and fixed with medical tape bandage (Figure 6).

**Figure 7:**
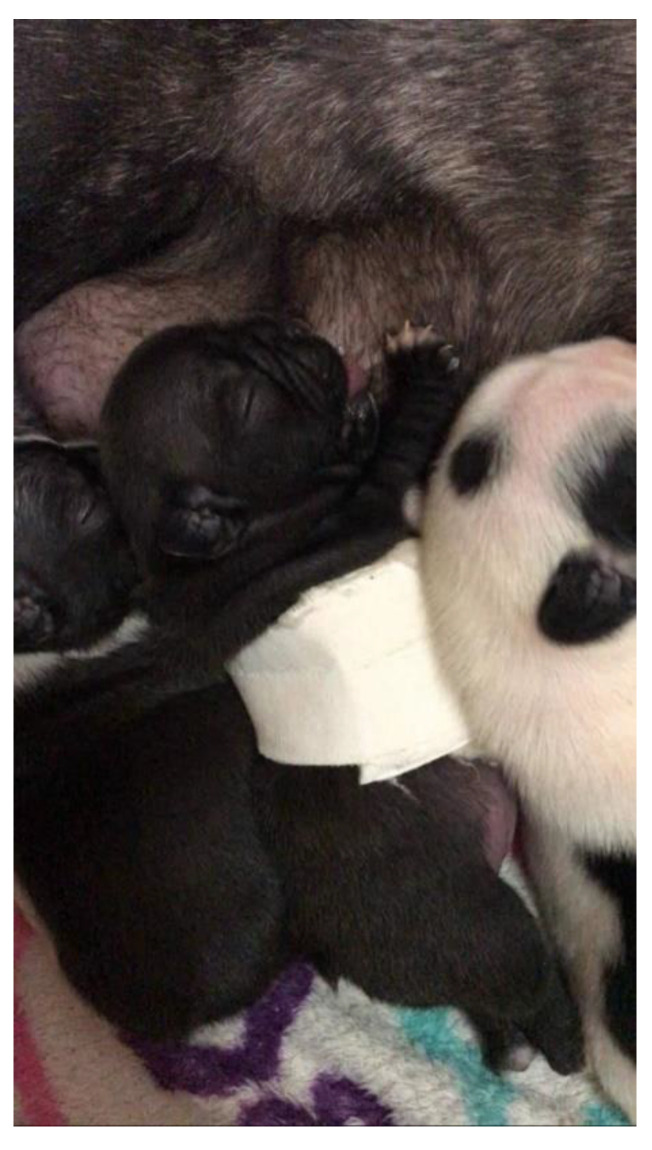
Patient nursing during splint use.

The puppy remained hospitalized for eight hours. After placing the splint, the patient was released home, as he already presented improvement of the respiratory pattern (clinical evaluation). After 24 h, the newborn already had a sucking reflex and fed normally on the mother. During treatment, the use of the splint did not hinder breastfeeding (Figure 7).

**Figure 8:**
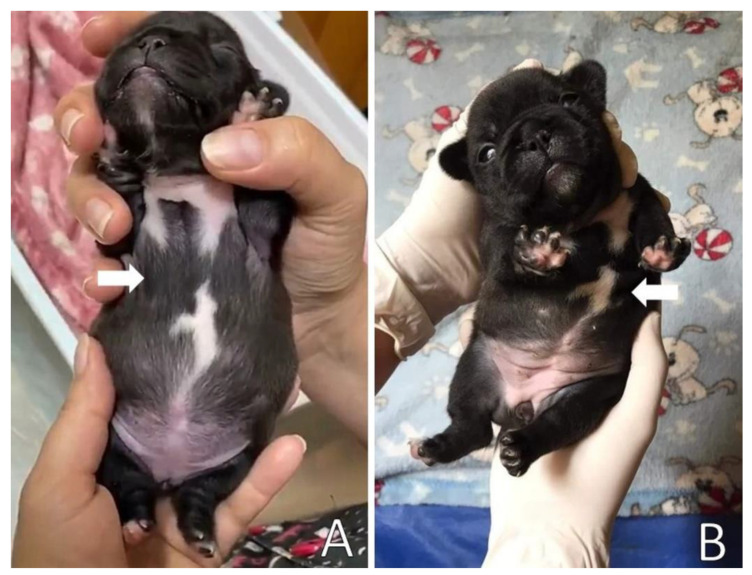
(**A**) Patient before splint management, presenting chest depression in the sternum bone region (arrow). (**B**) Patient after splint removal presenting chest with the absence of ventrodorsal narrowing in the sternal region (arrow) after noninvasive treatment.

On the return, eight days after the beginning of management, chest remodeling was observed, with the thorax correctly aligned, with absence of depression in the sternum bone region (Figure 8) and absence of respiratory distress. Video S2 demonstrates the respiratory pattern without alterations, with absence of substernal retraction during inspiration.

**Figure 9:**
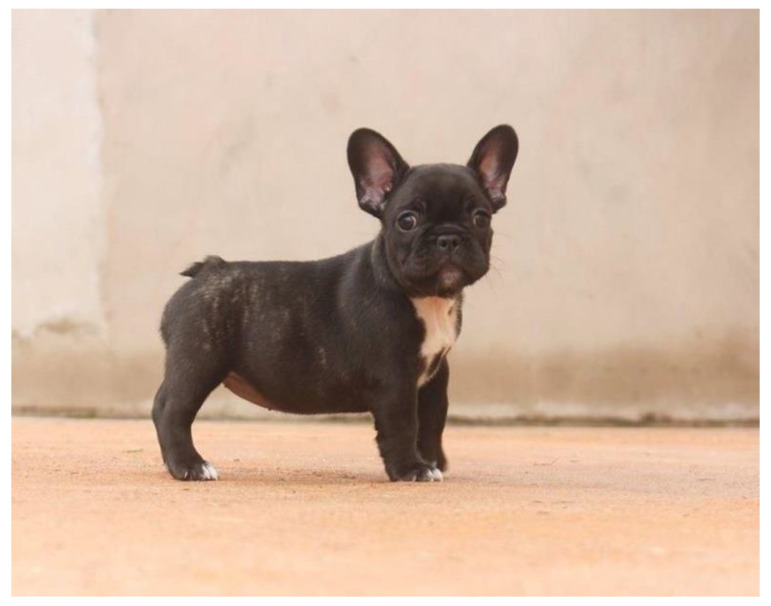
Puppy at two months old.

A new X-ray was requested for a detailed evaluation of the rib cage for a follow-up of the pulmonary condition and cardiac evaluation. However, the tutor chose not to perform the requested exams. Periodic evaluations and the future castration of the patient were recommended, as well as of parents and littermates. Figure 9 demonstrates the healthy puppy at two months of age. Currently, all puppies in the litter remain healthy, and all have been spayed or neutered.

### 2.2. Case 2—Paper Box Splint on Chest

A three-day-old male American bully dog was treated at the UNESP Veterinary Hospital, Botucatu, Brazil, with a history of dyspnea and cyanosis for two days. At birth, the pup was diagnosed with cleft palate (secondary palatoschisis, involving hard and soft palate, with shallow depth), did not ingest colostrum, and was fed exclusively with a commercial substitute of breast milk (Support Milk Dog^®^) by an orogastric tube (using urethral probe number 06), at a volume of 3 mL/100 g of weight and temperature of 37 °C every three hours. The newborn was separated from the mother and littermates on the day of birth and was kept in a plastic box with blankets and heated with a thermal bag.

Regarding maternal information, it was a primiparous female, at 12 months of age, fed a super-premium commercial diet, vaccinated, and dewormed, without historic diseases during pregnancy. No drugs were administered during the gestational phase. It was reported that the pregnancy came from inbreed mating between siblings of the same litter. The dog underwent elective cesarean section at approximately 62 days of gestation, with the birth of 10 puppies. The estimate of gestational age was determined by measuring fetal diameters on maternal ultrasound: biparietal (cephalic) diameter and thoracic diameter. No alterations were observed in the other neonates.

**Figure 10:**
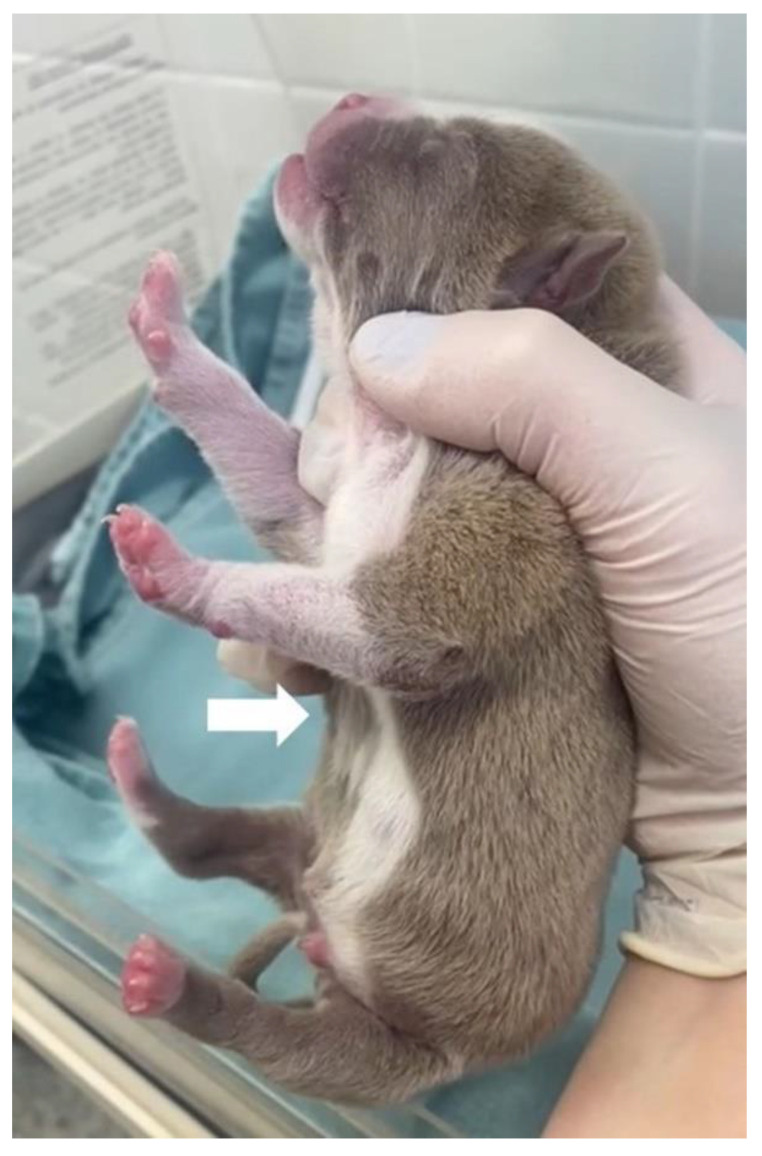
Ventrodorsal narrowing in the sternal region (arrow).

On physical examination, the patient presented dyspnea, substernal retraction during inspiration (Video S3), cyanosis during manipulation, HR 255 bpm, FR 30 mpm, body temperature 36.8 °C, glycemia 151 mg/dL, weak suction reflexes and breast search, strong vestibular straightening reflex, body weight 318 g, was normohydrate and without changes in cardiopulmonary auscultation. In the evaluation of the thorax, depression was observed in the caudal region of the sternum bone, and ventrodorsal narrowing was observed (Figure 10).

**Figure 11:**
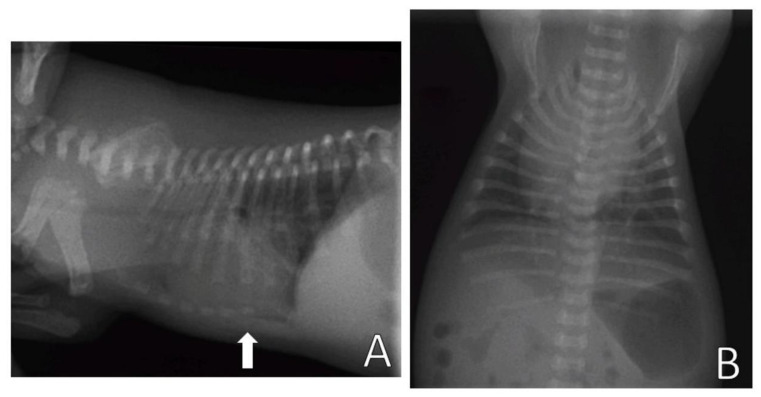
Chest X-ray. (**A**) Right lateral position, demonstrating ventrodorsal deviation of the sternum bone (arrow). (**B**) Ventrodorsal position, demonstrating rounded cardiac silhouette and right cardiac deviation.

Complementary tests, hemogram and chest X-ray, were performed. Blood (0.4 mL) from the jugular vein was collected for the hemogram, which was within the reference standards for age. The chest X-ray (Figure 11) was performed in the right lateral and ventrodorsal positions, observing a ventrodorsal deviation of the sternum and diagnosing pectus excavatum. The frontosagittal and vertebral indices corresponded to 1.7 cm (≤2 cm) and 10 cm (>9 cm), respectively, classifying the pectus as mild grade. In addition, a rounded cardiac silhouette (globose aspect) and right cardiac deviation were observed.

The treatment was performed with oxygen therapy per mask, with 100% oxygen for 10 min, and the sublingual administration of aminophylline 24 mg/mL in a volume of 0.2 mL/100 g of weight. As the neonate did not ingest colostrum, fresh frozen plasma was given subcutaneously from an adult dog, healthy and vaccinated, as a source of passive immunity, in a volume of 2 mL/100 g of weight [21,22]. Feeding by orogastric tube was indicated until later surgery to correct the palatoschisis.

**Figure 12:**
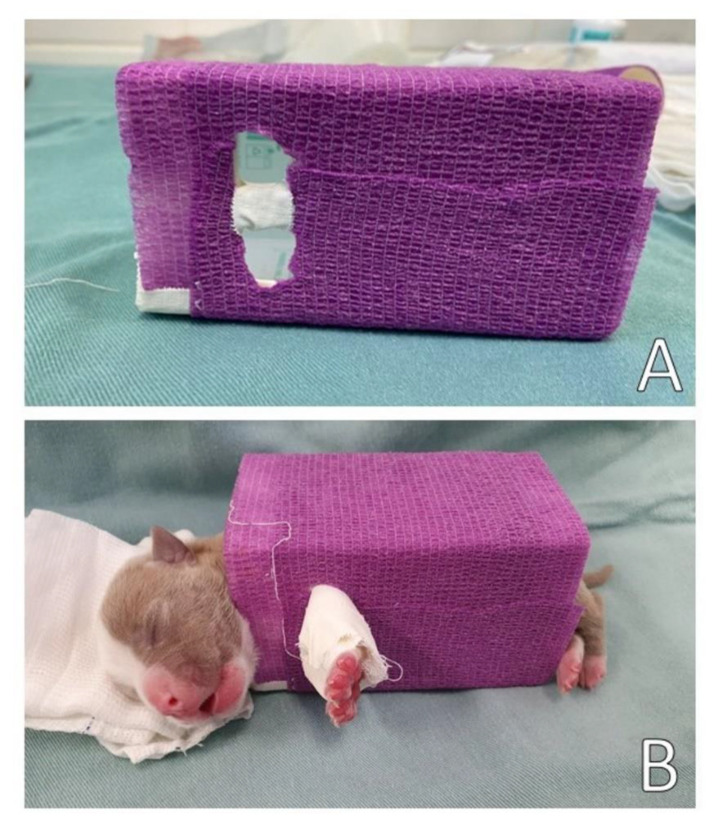
Box management. (**A**) Resistant paper box with space to insert and immobilize the previous members. (**B**) Puppy immobilized in the lateral decubitus position.

Corrective management of pectus excavatum was performed by positioning the neonate in lateral decubitus inside a resistant paper box [23]. In this case, for a neonatal puppy of 318 g, a medicine box was used (in a horizontal position), with dimensions of 6 cm high, 6 cm wide, and 11.5 cm long (Figure 12), aiming at the lateral compression of the thorax on surfaces so that the frontal region of the thorax would return to its normal positioning.

The box was coated with an elastic bandage, promoting greater stiffness. A space was made in the box to insert and immobilize (with gauze and medical tape) the thoracic limbs of the newborn to keep it immobile in lateral decubitus, avoiding its movement and consequent ventral compression of the thorax. After finishing the splint and bandage, the patient was released home. The lateral decubitus change occurred every two hours. The neonate was fed by an orogastric tube during the use of the box and was positioned in ventral decubitus for feeding.

**Figure 13:**
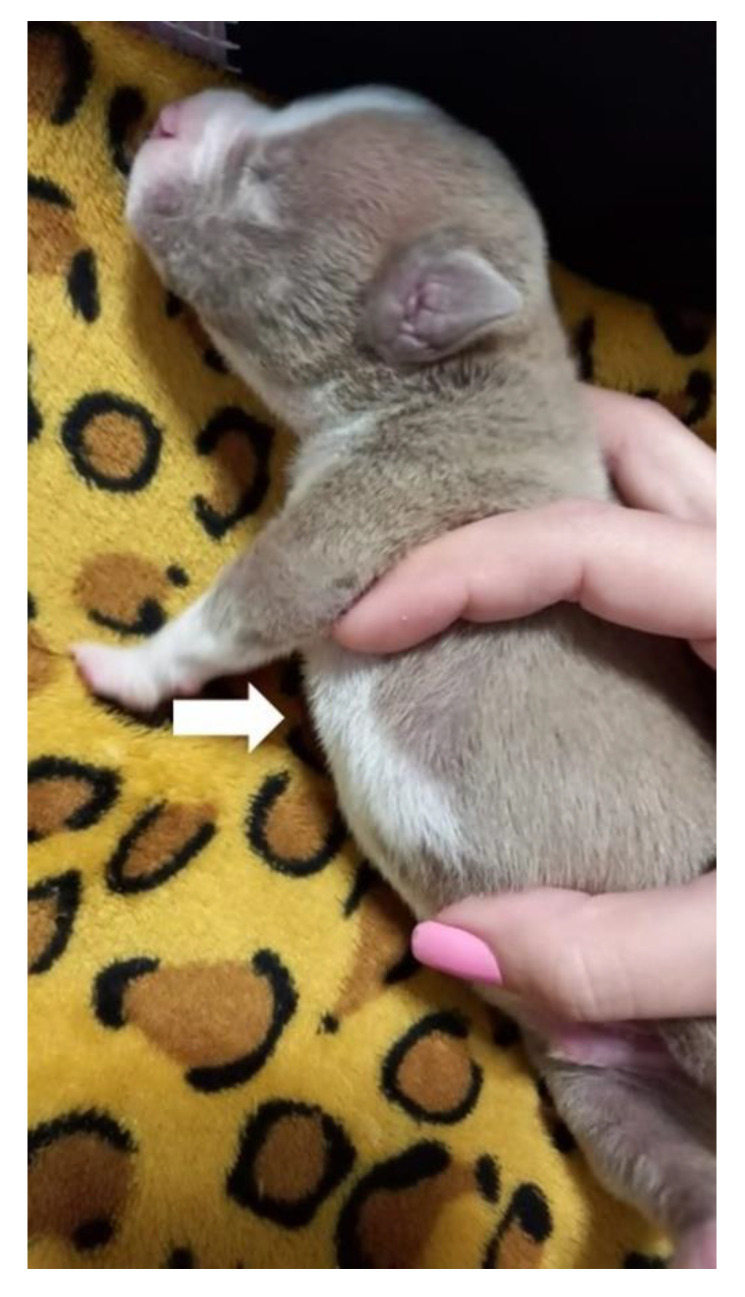
Patient after splint removal. Thorax presenting absence of ventrodorsal narrowing in the sternal region (arrow) after noninvasive treatment.

On the return, six days after the beginning of management, the neonate presented a chest wall without alterations, observing the remodeling of the thorax, absence of depression in the sternum region (Figure 13), regular respiratory pattern, absence of substernal retraction during inspiration (Video S4) and pink mucous membranes.

**Figure 14:**
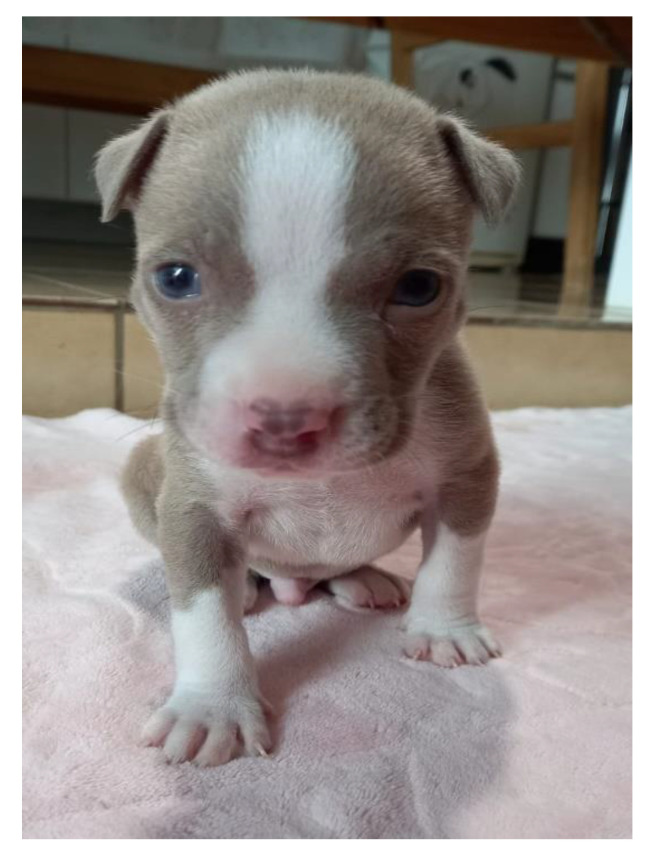
Puppy at 30 days old.

A new X-ray was requested for chest evaluation, but the tutor chose to perform the examination later. Periodic evaluations and future castration of the patient were recommended, as well as of parents and littermates. The puppy developed normally, and no chest deformations were observed. Figure 14 shows the healthy puppy at 30 days of age.

## 3. Discussion

The occurrence of malformations in dogs is attributed to prenatal factors, related to genetic causes, which may be hereditary, or to maternal exposure to teratogenic agents during pregnancy, such as toxins, radiation, chemical agents, infectious diseases, mechanical influences or drugs [24,25,26]. In the history and anamnesis of the cases described, there was no report of maternal exposure to teratogenic agents. However, in case 1, the kennel had a history of diagnosis of pectus excavatum in the litter of a female of the same family. Thus, the malformation of the French Bulldog puppy may be related to genetic factors of the kennel breed lineage. In humans and animals, approximately 40% of individuals with pectus excavatum have a first-degree member of the family affected by the deformity [27,28,29]. Moreover, in dogs, it is known that the highest incidence of malformations, approximately 84.4%, is observed in purebred puppies [24] and that brachycephalic breeds have a higher predisposition to manifest pectus excavatum, with a prevalence of 44% [3], indicating that this poor formation is related to genetic components in these breeds [4].

In case 2, the neonate came from consanguineous mating between siblings of the same litter. The main effect of consanguinity (or inbreeding) is the loss of genetic variability and increased homozygosis, which can lead to the manifestation of deleterious genes. The consequence of the reproductive process with this degree of familial relationship is that both carry genes related to malformations, and when they mate, they increase the chance of transmission of these replicas to their young, with potential manifestation of these deformities in the litter [24,30,31]. Therefore, as genetic defects can be inherited from one or both parents, being commonly described in purebred puppies [24,26], and pectus excavatum has a high prevalence in brachycephalic breeds, it is likely that the cause of malformation in the American Bully puppy is also related to genetic factors.

In puppies, pectus excavatum is present at birth and is detectable immediately after delivery or in a few days [1,15]. It is important to perform a thorough physical examination of newborn dogs for the presence of malformations and to guide the tutor or breeder in evaluating the presence of some abnormality in the inspection of litters. Early diagnosis is essential for rapid treatment and a greater chance of survival of patients, since this deformity may be progressive, with the worsening of clinical signs. Depression of the sternum bone in the pectus is identifiable and palpable by physical examination; however, it is important to perform imaging tests, as they help in the classification of the degree of impairment of the defect and in the diagnosis of cardiac and respiratory tract abnormalities associated with this malformation. For example, the globose aspect of the heart may be associated with cardiac compression caused by pectus excavatum, associated congenital heart diseases, or due to altered cardiac positioning, which may cause the heart to appear radiologically enlarged [15]. Due to the cardiorespiratory alterations of the patients in this report, the continued evaluation by imaging tests, such as chest X-ray and echocardiogram, was recommended.

An evident clinical sign in the pups of this report was substernal retraction during inspiration (retraction of the abdomen just below the sternum). Retractions can be observed in newborns and children, as they have a more flexible thorax than adults [32]. Thoracic retractions indicate that the patient is having difficulty breathing (high intrathoracic pressure required during inspiration to expand the lungs), and the substernal type is associated with the degree of mild to moderate respiratory difficulty [32,33,34]. It is important to include pectus excavatum as a differential diagnosis in neonatal puppies with dyspnea and substernal retractions, especially in brachycephalic dogs. In addition, because pectus excavatum is often underdiagnosed, pneumonia that does not respond adequately to treatment should be further investigated.

Leukocytosis with left deviation associated with neutrophilia and slight absolute lymphocytosis was observed in the blood count of case 1. This alteration may be associated with the pneumonia presented by this puppy, associated with a possible secondary bacterial infection. The neonatal blood count should be interpreted based on the reference parameters for the age (in weeks) of the patients, as described by Bird, 2011 [35] and Von Dehn, 2014 [36]. Antibiotic therapy with ceftriaxone, a broad-spectrum beta-lactam drug considered safe for newborn dogs, was instituted. Cephalosporins and penicillins are frequently used classes of antibiotics recommended for the treatment of neonatal infections [37,38,39].

The choice between conservative or surgical treatment can be dictated by the severity of the defect, age of the animal, stiffness of the costal arches and capacity of retraction of the sternal defect [40]. The use of corrective splints as a noninvasive treatment was effective in the present cases (mild degrees) and is a feasible option to be performed in newborn dogs. This occurs due to the flexibility of the costal cartilages and sternum of the pups, which facilitates thoracic remodeling [15]. Young animals with mild pectus excavatum may present a good response to the use of corrective thoracic bandages without surgical intervention and only with conservative management. However, the lateral-medial compression of the thorax should not be used in more severe cases, as it may exacerbate dorsal intrusion of the sternum into the thoracic cavity. In these cases, surgery is indicated [15,40]; an ostectomy of a section of the costal cartilages can be performed to allow the sternum to be realigned, and bone plaque can be used to keep the sternum in this position. Furthermore, splints with circular sutures on the sternum can be used. The sutures are tied to the splint to keep the thorax in a normal position and kept in place for three to four weeks [15,17,41].

In a case report of pectus excavatum in an English Bulldog neonatal puppy, the use of the noninvasive PVC pipe splint was also effective, demonstrating the general clinical improvement of the patient, anatomical conformation of the thorax close to normal, and restoration of the appropriate respiratory pattern [2]. However, the management of the box in the thorax, demonstrated in this report, is unprecedented and has not yet been described in the literature.

Regarding the choice of which splint to use, although the two types are effective, in our routine neonatal care, we observed that puppies with intense respiratory distress benefit from the use of the box compared to the PVC pipe, since in the management of the pipe in the chest, some animals may become uncomfortable due to greater lateral compression. We suggest testing the management in which the puppy will become more comfortable, observing the absence of crying, restlessness or exacerbation of dyspnea. The length of stay with the splint is also variable; in our care routine, we already observe complete remodeling of the chest in two to eight days. For the removal of the splint, the thorax should be inspected daily, removing the splint to observe the absence of depth of the sternum region and improvement of the respiratory pattern.

Because it is a hereditary malformation, it is not recommended that animals diagnosed with pectus excavatum be used for reproductive purposes, and spay/neuter is recommended, even if they do not show clinical signs [6,15]. This information was emphasized to the case tutors, as well as oriented about avoiding the continuity of the parents in the reproductive process, recommending the spay or neuter of these and animals with a history of pectus excavatum in their litters.

Congenital malformations have been diagnosed more frequently in newborn dogs [24]. It is important that the veterinarian has knowledge about the clinical or surgical approach of malformations to indicate the best corrective conduct, as well as to explain to the tutor or breeder the possibility of managing/treating each defect.

As preventive measures, care should be taken in the choice of parents, avoidance of inbreeding and animals with a history of genetic problems or defects, as well as maternal exposure to teratogenic factors during pregnancy. Prenatal care is essential to prevent malformations and reduce mortality rates in neonatal puppies [24,31,39].

## 4. Conclusions

Management with corrective splints (PVC pipe and box) is effective for the conservative treatment of pectus excavatum in neonatal puppies, resulting in the remodeling of the thorax and improvement of the respiratory pattern. However, more severe cases may need corrective surgery. Each case should be evaluated individually. In the cases presented, pectus excavatum was probably related to genetic factors associated with racial predisposition. In dogs, this is a progressive condition with cardiorespiratory involvement and should be diagnosed early and treated as soon as possible to avoid the evolution of clinical signs and increase the chance of neonatal survival.

## Figures and Tables

**Table 1:** Frontosagittal and vertebral thoracic indices in dogs of nonbrachycephalic breeds, brachycephalic breeds and cats [16].

Indices	Nonbrachycephalic Breeds	Brachycephalic Breeds	Cats
Frontosagittal (cm)	0.8–1.4	1–1.5	0.7–1.3
Vertebral (cm)	11.8–19.6	12.5–16.5	12.6–18.8

**Table 2:** Classification of pectus excavatum in dogs and cats based on frontosagittal and vertebral thoracic indices [16].

Pectus Excavatum	Indices (cm)	
	Frontosagittal	Vertebral
Mild	≤2	>9
Moderate	2–3	6–8.99
Severe	>3	<6

## Data Availability

Not applicable.

## Supplementary Materials

The following supporting information can be downloaded at: https://www.mdpi.com/article/10.3390/ani13050906/s1, Video S1: Neonate with pectus excavatum presenting dyspnea and substernal retraction during inspiration; Video S2: Neonate presenting respiratory pattern without alterations, with absence of substernal retraction during inspiration after management of the pipe splint; Video S3: Neonate with pectus excavatum presenting dyspnea and substernal retraction during inspiration; Video S4: Neonate presenting regular respiratory pattern, with absence of substernal retraction during inspiration after management of the splint of the box.
